# Supplementary material for: Causal Relationship between Aging and Anorexia Nervosa: A White-Matter-Microstructure-Mediated Mendelian Randomization Analysis
Source: Biomedicines. 2024 Aug 16;12(8):1874. doi: 10.3390/biomedicines12081874 (PMC11351342; doi:10.3390/biomedicines12081874)
Supplement: Supplementary file 1 [file biomedicines-12-01874-s001.zip › Supplementary Figure/Figure S4/Singe SNP plot of MR analysis for TL to ubm-b-1973.pdf]

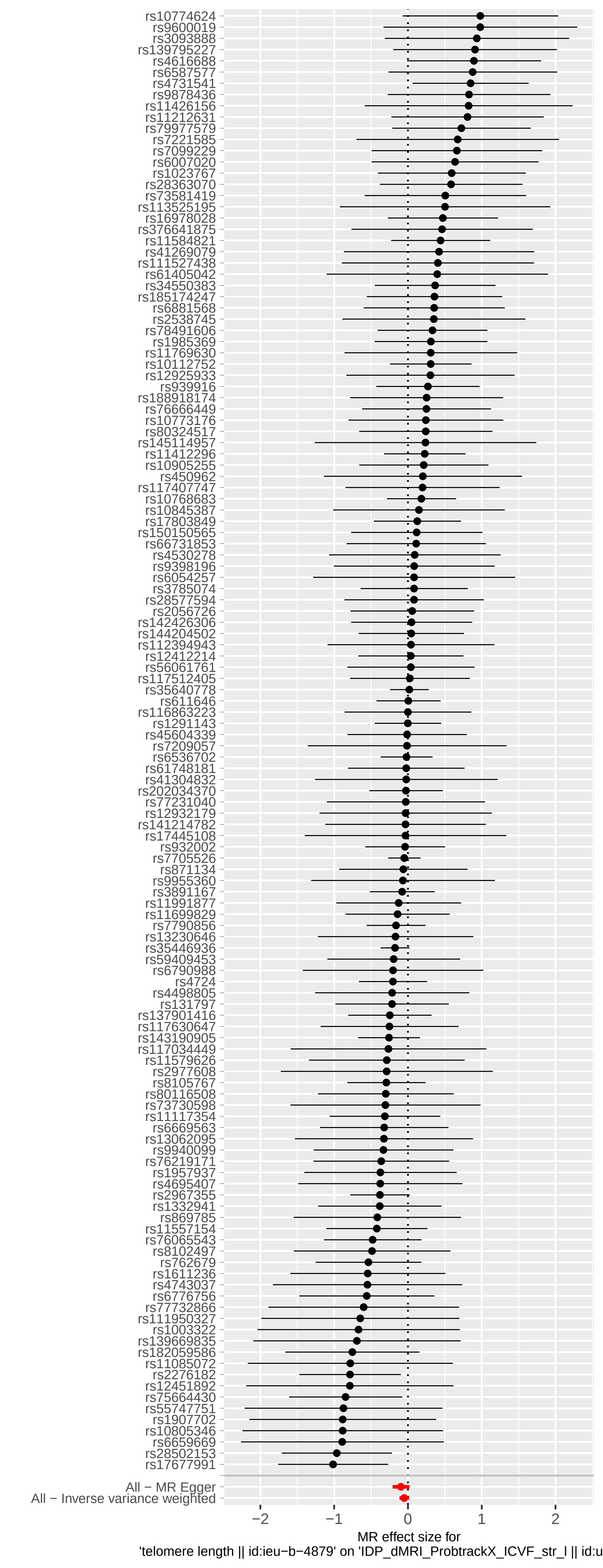
rs10774624
rs9600019
rs3093888
rs139795227
rs4616688
rs6587577
rs4731541
rs9878436
rs11426156
rs11212631
rs79977579
rs7221585
rs7099229
rs6007020
rs1023767
rs28363070
rs73581419
rs113525195
rs16978028
rs376641875
rs11584821
rs41269079
rs111527438
rs61405042
rs34550383
rs185174247
rs6881568
rs2538745
rs78491606
rs1985369
rs11769630
rs10112752
rs12925933
rs939916
rs188918174
rs76666449
rs10773176
rs80324517
rs145114957
rs11412296
rs10905255
rs450962
rs117407747
rs10768683
rs10845387
rs17803849
rs150150565
rs66731853
rs4530278
rs9398196
rs6054257
rs3785074
rs28577594
rs2056726
rs142426306
rs144204502
rs112394943
rs12412214
rs56061761
rs117512405
rs35640778
rs611646
rs116863223
rs1291143
rs45604339
rs7209057
rs6536702
rs61748181
rs41304832
rs202034370
rs77231040
rs12932179
rs141214782
rs17445108
rs932002
rs7705526
rs871134
rs9955360
rs3891167
rs11991877
rs11699829
rs7790856
rs13230646
rs35446936
rs59409453
rs6790988
rs4724
rs4498805
rs131797
rs137901416
rs117630647
rs143190905
rs117034449
rs11579626
rs2977608
rs8105767
rs80116508
rs73730598
rs11117354
rs6669563
rs13062095
rs9940099
rs76219171
rs1957937
rs4695407
rs2967355
rs1332941
rs869785
rs11557154
rs76065543
rs8102497
rs762679
rs1611236
rs4743037
rs6776756
rs77732866
rs111950327
rs1003322
rs139669835
rs182059586
rs11085072
rs2276182
rs12451892
rs75664430
rs55747751
rs1907702
rs10805346
rs6659669
rs28502153
rs17677991
All − MR Egger
All − Inverse variance weighted
−2
−1
0
1
2
MR effect size for
'telomere length || id:ieu−b−4879' on 'IDP_dMRI_ProbtrackX_ICVF_str_l || id:u
